# Weak Spatial and Temporal Population Genetic Structure in the Rosy Apple Aphid, *Dysaphis plantaginea*, in French Apple Orchards

**DOI:** 10.1371/journal.pone.0021263

**Published:** 2011-06-20

**Authors:** Thomas Guillemaud, Aurélie Blin, Sylvaine Simon, Karine Morel, Pierre Franck

**Affiliations:** 1 Equipe “Biologie des Populations en Interaction”, UMR 1301 I.B.S.V. INRA-UNSA-CNRS, Sophia Antipolis, France; 2 UR1115 Plantes et Systèmes de Culture Horticoles, INRA, Avignon, France; 3 UE695 Recherche Intégrée, INRA, Domaine de Gotheron, Saint-Marcel-lès-Valence, France; University of Florida, United States of America

## Abstract

We used eight microsatellite loci and a set of 20 aphid samples to investigate the spatial and temporal genetic structure of rosy apple aphid populations from 13 apple orchards situated in four different regions in France. Genetic variability was very similar between orchard populations and between winged populations collected before sexual reproduction in the fall and populations collected from colonies in the spring. A very small proportion of individuals (∼2%) had identical multilocus genotypes. Genetic differentiation between orchards was low (*F*_ST_ *<*0.026), with significant differentiation observed only between orchards from different regions, but no isolation by distance was detected. These results are consistent with high levels of genetic mixing in holocyclic *Dysaphis plantaginae* populations (host alternation through migration and sexual reproduction). These findings concerning the adaptation of the rosy apple aphid have potential consequences for pest management.

## Introduction

The rosy apple aphid *Dysaphis plantaginea* (Hemiptera: Aphididae) is one of the most serious pests of apple trees in Europe [1] and North America [2]. It causes fruit deformation and severe leaf-curling [3], distorts shoots, reduces flower formation and slows tree growth [4].

In commercial apple tree orchards, the damage caused by even very low densities of aphids may decrease the commercial value of the crop. This economic loss justifies aphid management techniques, based principally on pesticide use. Recommendations generally suggest the use of several pesticide treatments in apple orchards: in early spring, before flowering and after flowering or in late summer [5]. The intensive use of chemical insecticides against *D. plantaginea* has resulted in an intense selection regime and the development of mechanisms of insecticide resistance in the field [6]. Alternative control strategies, such as the application of organic pesticides (neem extract or potassium soap [5]), the use of repellent or barrier-effect products (kaolin [7], [8], [9]), biological control [10], [11], [12], and plant resistance [13], [14], [15], [16], are being developed and tested.

Whatever the pest management strategy applied, the likelihood of developing resistance to management depends on the ecological characteristics of the target species: its migration capability, sexual reproduction and clonal multiplication determine, at least in part, its genetic variability and, thus, its capacity to adapt to control measures. An analysis of genetic variation in the *D. plantaginea* population may therefore provide essential information about these crucial ecological parameters.

The life cycle of *D. plantaginea* almost certainly has profound consequences for its genetic variability. Like many aphid species, *D. plantaginea* has a cyclic parthenogenetic (or holocyclic) life cycle [17], [18]. In late summer and fall, cyclically parthenogenetic aphids give birth to gynoparae (precursor forms of sexual females), followed by winged males. Both fly from the herbaceous secondary host plant, *Plantago*, to the primary host, apple trees, where the gynoparae give birth to sexual females [19]. Mating occurs on apple and sexual females lay eggs that hatch by the beginning of spring. During late spring and early summer, after 3 to 4 (maximum 6) parthenogenetic generations, winged morphs are produced that migrate from the primary to the secondary host on which about 3 to 8 successive parthenogenetic generations occur [19]. Thus, due to the annual host alternation, two large migration events take place in biological cycle of *D. plantaginea*, in the fall and spring.

In many species, cyclic parthenogenetic populations coexist with obligate parthenogenetic populations [20], [21]. In such populations, the aphids have lost the ability to reproduce sexually and remain on herbaceous plants throughout the year. According to Lathrop [22], the rosy apple aphid does not occur on plantain during winter in colder parts of the USA. However, “in the mild climate of western Oregon, overwintering on plantain as well as apple is the rule” [22]. This suggests that this species displays variation in reproductive modes, with cyclic parthenogenetic populations coexisting with obligate parthenogenetic populations. However, we are not aware of any other study demonstrating such a polymorphism in *D. plantaginea*.

Cyclic parthenogenetic aphids would be expected to display high levels of genotypic variability, due to the recombination occurring during sexual reproduction [21], [23], [24]. However, drift and/or selection may strongly decrease neutral genetic variability during successive parthenogenetic generations after egg hatching on apple and on secondary hosts, due to the absence of recombination and the rapid rate of increase during clonal reproduction as shown in the peach-potato aphid *Myzus persicae* [24], [25]. During this clonal phase, genetic signs of parthenogenesis may accumulate: linkage disequilibrium (LD), Hardy-Weinberg (HW) disequilibrium, and decrease in multilocus genotype diversity [23].

Little is known about the genetic diversity of the *D. plantaginea* species. The only data available are the preliminary results obtained by Salomon *et al*. [26], who reported high levels of genetic variability in a single apple orchard, based on an analysis of microsatellite genetic markers previously developed by Harvey *et al*. [27] for this species. We therefore know little about the effects of the succession of sexual and asexual reproduction on the genetic variability of this species or those of the major migration events occurring during host shift.

The aim of this study was to determine whether the complex mode of reproduction, with a single sexual generation and successive clonal generations, and host shift-related migration events affected genetic variation in this species. In other words, we evaluated the geographic scale over which *D. plantaginea* populations function and possible decreases in the genetic variation of *D. plantaginea* on apple due to cyclic parthenogenesis.

More specifically, we used a geographic and temporal sampling scheme and highly polymorphic genetic markers (microsatellite) data to address the following questions: (i) What degree of genetic variability does the rosy apple aphid display at the national scale (over the whole of France)? (ii) Is there any genetic differentiation between populations of *D. plantaginea* and at what level (regions, orchards, apple cultivars) can this differentiation be detected? (iii) Are the genetic diversity and geographic population structure of *D. plantaginea* stable at different parts of the life cycle and in different years?

## Materials and Methods

### Sample collection

**Figure 1:**
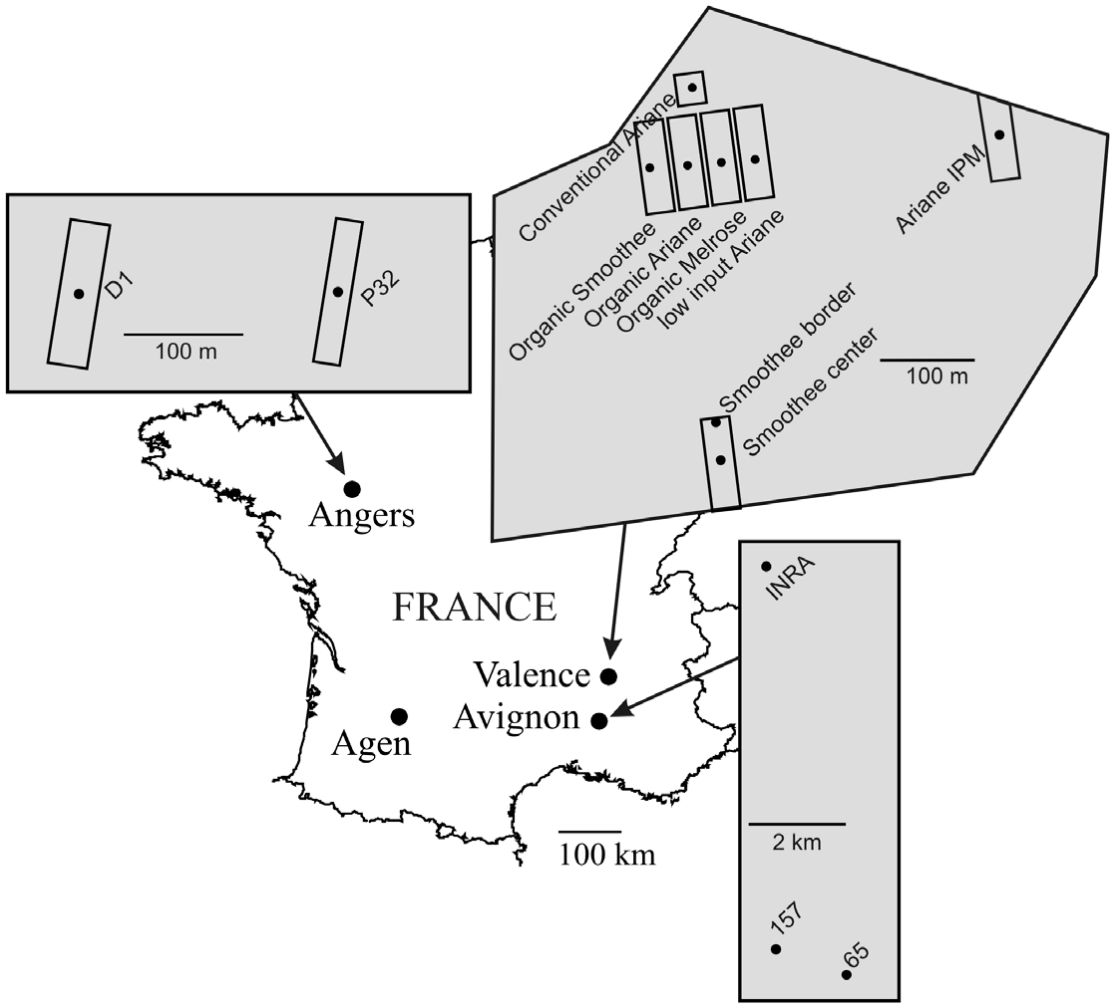
Locations of the samples of *Dysaphis plantaginea* used in this study. Sampling periods are indicated.

Samples were collected according to a geographic and temporal scheme in experimental apple orchards belonging to INRA institute. Here an orchard is defined as a field of apple trees with a given management strategy and a specific tree cultivar. The term “sample” refers to as a group of aphids collected during a specific season and at a specific position in a given orchard. No specific permission was required to sample aphids in these orchards. They were collected at one location in north-western France (near Angers), one location in south-western France (near Agen), and two locations in southern France (near Avignon and Valence) (Figure 1). Depending on the location, aphids were sampled at one (Agen), two (Avignon, Angers) or three (Valence) different periods of the aphid life cycle, in fall 2006 and 2007, and in spring 2007 (see Table S1). Furthermore, at Avignon, Valence and Angers, samples were taken from different orchards at the same time (Table S1). The distances between these orchards were as follows. At Valence, the various orchards that were sampled were located from within a circle with a radius of 250 m. The Smoothee1 orchard sample was located about 350 to 450 m from the other orchard samples, the Conventional Ariane orchard sample was about 300–450 m from the other samples, and the remaining orchard samples were located about 10 to 100 meters apart. At Valence, samples were collected on different apple cultivars (Smoothee, Melrose and Ariane) under organic management, but also from different plants of the same cultivar (Ariane) grown under organic, low-input and conventional pest management regimes (i.e. organic-registered for the organic system, minimized for the low-input system and supervised for the conventional system). Two locations (center and border) in Smoothee1 orchard in Valence were sampled in autumn 2006 to test for micro-geographic genetic structure that would not depend on tree cultivars and management strategies. At Angers, the two orchards sampled, P32 and D1, were located 500 meters apart. Finally, at Avignon, orchards 65 and 157 were located 2.5 km apart, each about 12 to 15 km from the INRA orchard. In the fall, winged gynoparae were sampled manually by branch tapping. In spring, individuals were collected by hand, with a small brush, with no more than one individual collected per colony and per tree on two sampling dates (May 8 and 23). Aphids were stored in absolute ethanol for DNA extraction.

### DNA extraction and microsatellite analysis

Template material for the amplification of microsatellites by PCR was prepared from individual aphids with the “salting out” rapid extraction protocol [28] and resuspended in 50 µl H_2_O. Eight microsatellite loci for *D. plantaginea* (DpL4, DpB10) [27], *Sitobion* species (S24, Sa4Σ, S3.43, S16b) [29], *Rhopalosiphon padi* (R5.29B) [29] and *Aphis fabae* (AF93) [30] were amplified in two separate multiplex PCRs. The first reaction amplified *DpL4*, *DpB10*, *S24* and *Sa4Σ*, and the second amplified *S3.43*, *AF93*, *R5.29B* and *S16b*. Both multiplex reactions were carried out with Qiagen multiplex PCR kits (Qiagen, Hilden, Germany), according to the manufacturer's instructions, in a final volume of 10 µl containing 1 µl of DNA template. The forward primer for each microsatellite was labeled with a fluorescent dye, to allow the detection of PCR products on an ABI 3100 DNA sequencer (Applied Biosystems, Foster City, CA). We used the following PCR program for both reactions: 95°C for 15 minutes, followed by 35 cycles of 30 s at 94°C, 90 s at 56°C, 1 min at 70°C, and 30 s at 60°C.

### Data analysis

Within-population genetic diversity was estimated by calculating the number of alleles per locus, and observed and expected heterozygosities calculated with GENEPOP ver. 4.0 [31], [32]. Exact tests for deviation from Hardy-Weinberg (HW) expectations, linkage disequilibrium and population differentiation were carried out with GENEPOP. A Mantel test of isolation by distance was also carried out with Genepop ver. 3.1 [31]. MICROCHECKER was used to detect the presence of null alleles at each microsatellite locus [33] and genotypic differentiation between pairs of populations (*F*_ST_) was corrected for null alleles as described by Chapuis *et al*. [34]. We compared the number of alleles per locus between population samples, by estimating allelic richness (*AR*) on the basis of minimum sample size, with the rarefaction method [35] implemented in Fstat 2.9.3 [36].

If more than one copy of the same multilocus genotype (MLG) was observed, the null hypothesis of the same MLG being obtained repeatedly by chance through sexual reproduction was tested with Genclone ver. 2.0 [37]. This test is based on calculation of the probabilities of obtaining MLGs from sexual events, taking into account the estimated *F_IS_* for the population.

Finally, the number of distinct populations (*K*) present in the set of samples was estimated with STRUCTURE [38]. This software was used to estimate Pr(*X*|*K*), the probability of the observed set of genotypes (*X*), conditional on the number of genetically distinct populations, *K*, for values of *K* between 1 and the number of samples. The program was run for 10^5^ iterations, preceded by an initial burn-in period of 2×10^4^ iterations. Three runs were performed for each value of *K*, to check that estimates of Pr(*X*|*K*) were consistent between runs. The posterior probabilities, Pr(*K*|*X*), were then calculated as described by Pritchard *et al*. [38].

For multiple tests of a single hypothesis and non orthogonal comparisons, we used Benjamini & Hochberg [39] and sequential Bonferroni [40] correction procedures, respectively, to correct significance levels.

## Results

### Within-population variability

We genotyped 532 individuals in total and found the level of genetic variation to be high. There were seven (locus *S3.43*) to 34 (locus *S24*) alleles per microsatellite locus. Within-population genetic variability was high, with mean numbers of alleles per locus (Na) of more than seven for samples with more than 15 individuals. Allelic richness (*AR*), calculated on a sample of at least 15 individuals for inter-population comparisons, was between 3.9 and 4.3 (mean *AR* = 4.14, SEM = 0.13), and revealed no difference in population variability between samples and between spring and fall (Friedman analysis of variance and Wilcoxon's signed rank test, *p*>0.05). Consistent with this, no heterogeneity of Nei's heterozygosity was detected (mean *He* = 0.63, SE = 0.03; Friedman analysis of variance and Wilcoxon's signed rank test, *p* = 0.41 and *p* = 0.32, for between-sample and between spring and fall comparisons, respectively). All samples displayed a heterozygote deficiency, with many genotypic compositions showing departure from HW equilibrium (Table S1). The instances of HW departure identified frequently involved the same three loci (*DPL4*, *DPB10* and *AF93*), suggesting the presence of null alleles at these loci. Loci *DPL4*, *DPB10* and *AF93* displayed departure from HW equilibrium eight, seven and five times, respectively, in a total of 26 significant per locus and per sample tests. MICROCHECKER suggested the existence of null alleles for *DPB10* and *DPB4*. No heterogeneity in the proportion of significant HW tests was found between samples or between spring and fall samples (Fisher's exact test on RxC contingency tables, *p*>0.05 for both tests). Accordingly, no heterogeneity in *F*_IS_ value was detected between samples or between spring and fall samples (Friedman analysis of variance and Wilcoxon's signed rank test on mean *F*_IS_ value per locus, p = 0.51 and p = 0.33, respectively). After removal of the *DPL4*, *DPB10* and *AF93* loci from the analysis, the general heterozygote deficiency remained and no heterogeneity was apparent between samples or between spring and fall (Friedman analysis of variance and Wilcoxon's signed rank test on mean *F*_IS_ value per locus, *p* = 0.72 and 0.89 respectively).

A very high level of multilocus genotypic variability was found. PCR amplification was unsuccessful in some cases. In total, 342 individuals were genotyped with no missing data, and 336 different multilocus genotypes (MLG) were detected in these individuals (ratio of the number of multilocus genotypes over the total number of individuals, *N*_MLG_ */N* = 0.98). Six MLGs were found in multiple copies. Each of these repeated MLGs was found in two individuals sampled from the same orchard on the same date: orchards 65 and 157 in fall 2006, orchards Bio Smoothee and Bio Melrose in Valence in spring 2007, and Agen in fall 2007. These repeated MLGs were probably generated by clonal rather than sexual reproduction (test of the null hypothesis of sexual recombination, *p*<8×10^−4^). Consistent with the extensive multilocus genotypic variability observed, an analysis of the genotypic disequilibrium between each pair of loci in each sample revealed very few cases of significant linkage. No heterogeneity in the number of significant LD was found either between samples, or between spring and fall (Fisher's exact test on RxC contingency tables, *p*>0.05 for both tests).

### Population differentiation

As most samples displayed heterozygote deficiency, we carried out exact tests of genotypic differentiation between samples only. All comparisons between parts of orchards or between orchards at the same location or at the same period were characterized by small *F*_ST_ values (<1%) and non significant differentiation tests (*p* = 0.078 and 0.25 at Angers, *p* = 0.15 and 0.47 at Avignon in fall 2006 and 2007 respectively, and *p* = 0.43 at Valence in fall 2007). Parts of orchards and orchards at the same location were therefore pooled by period for analyses of regional genetic differentiation (Table 1).

**Table 1:** Regional and temporal differentiation of *Dysaphis plantaginea* samples in France.

		Fall 2006			Spring 2007		Fall 2007		
		Angers	Avignon	Valence	Valence	Agen	Angers	Avignon	Valence
Fall 2006	Angers	-	0.002	0.008			0.005		
	Avignon	0.018*	-	0.009				0.008	
	Valence	0.001**	3×10^−4^**	-	0.002				−0.002
Spring 2007	Valence			0.023	-				0.001
Fall 2007	Agen					-	0.006	0.026	0.012
	Angers	0.004*				0.006*	-	0.016	0.003
	Avignon		8×10^−4^**			10^−5^**	3×10^−4^**	-	0.024
	Valence			0.3	0.56	0.035*	0.223	10^−5^**	-

Pairwise estimates of *F*_ST_ are above the diagonal and the *p*-values of genotypic differentiation exact tests are shown below the diagonal. * and ** after *p*-values indicate that the tests were significant before and after Bonferroni correction, respectively. Only pertinent comparisons (i.e. between periods at the same sites or between sites during the same period) are shown.

As null alleles were suspected for several loci, we also performed an analysis taking these null alleles into account [34]. We found the same absence of differentiation between samples from the same location, with the exception of two orchards in Avignon sampled in 2006 (165 and 57, *p* = 10^−3^). As the level of genetic differentiation was very low (*F*_ST_ = 4.3×10^−3^) we decided to pool the samples from each location.

Significant, but weak (*F*_ST_<1%) genotypic differentiation was detected between Angers, Avignon and Valence in fall 2006 (Table 1). In fall 2007, significant moderate levels of differentiation were observed between Avignon and other locations (*F*_ST_∼2%). A low level of differentiation was found between Agen and Angers or Valence (*F*_ST_∼1%) and no differentiation was detected between Angers and Valence. The same overall pattern was observed if null alleles were taken into account: significant, but low to moderate levels of differentiation between locations.

Only low to very low levels of differentiation were found between samples from the same location collected at different time periods. Almost no difference was found between samples collected at Valence in fall 2006, spring 2007 and fall 2007 (although the differentiation between fall 2006 and spring 2007 was of borderline significance, *p* = 0.023, *F*_ST_ = 0.002). Comparisons between fall 2006 and 2007 for each location revealed significant but weak (in the case of Angers and Avignon, *p*<4×10^−3^, *F*_ST_ = 0.005 and 0.008, respectively) and non significant (in the case of Valence, *p* = 0.3, *F*_ST_ = −0.002) differentiation.

Very similar results were obtained when null alleles were taken into account. In this case, significant differentiation was detected in all comparisons other than that between fall 2006 and fall 2007 at Valence. No isolation by distance was detected between the 16 samples with more than 15 individuals (Mantel test, *p* = 0.153).

A Bayesian analysis of population structure grouped all individuals together in a single population, regardless of their location and sampling period (*P*(*K* = 1|*X*) = 1). This was true for the default model (admixture and correlated allele frequency), but also for the admixture and independent allele frequency model. Models without admixture gave inconsistent results (*P*(*K* = 2|*X*) = 1 and *P*(*K* = 14|*X*) = 1 for the correlated and independent allele frequency models, respectively). Evanno's ΔK [41] also gave inconsistent results for the models without admixture (K = 5 and K = 2 for the correlated and independent allele frequency models, respectively).

## Discussion

### Considerable variability and no evidence for obligate parthenogenesis

In this study, we analyzed the genetic structure of populations of the rosy apple aphid, *D. plantaginae*, collected from its primary host. The goal was to characterize, for the first time, the genetic variability of this aphid, and to evaluate the impact of three evolutionary forces potentially affecting this variation: drift, migration and selection. Rosy apple aphid populations collected from apple trees in four regions of France displayed extensive genetic variation. In particular, a very high degree of genotypic diversity was observed, with almost all individuals genetically different from each other. This was true for all locations and sampling periods. This result confirms and extends the findings of Solomon *et al.* [26], who were the first to report high levels of genetic variability in *D. plantaginea* sampled from apple orchards.

The rosy apple aphid is thought to be a cyclic parthenogenetic species, with a single sexual generation and many asexual generations. It is unknown whether this species displays polymorphism in its mode of reproduction, with the coexistence of obligate parthenogenetic and parthenogenetic individuals, as in many other aphid species [42]. The mode of reproduction has consequences for the genetic variation of populations [43], and this topic has been particularly well studied in aphids [44]. In the case of holocycly, two antagonistic effects occur. Asexual generations (reproducing by mitotic parthenogenesis in this species) are expected to generate individuals with an identical genetic background, with mutations as the only source of variation. The occurrence of such asexual generations also leads to systematic linkage disequilibrium (LD) and departure from HW equilibrium. By contrast, (panmictic) sexual generation disrupts inter-locus associations, resulting in each individual being genetically different from all others. It also re-establishes HW equilibrium within a single generation and decreases LD. Note that, in the long term, obligate parthenogenesis (parthogenesis as the only form of reproduction) tends to lead to excess heterozygosity due to the accumulation of mutations without recombination [44].

In French populations of the rosy apple aphid collected from its primary host we found neither general LD, nor a global excess of heterozygotes. We found extensive multilocus genotypic variability. These genetic signals provide evidence of sexual reproduction, supporting the hypothesis that the populations collected from apple trees in the spring and fall are holocyclic. This is consistent with what is known of the lifecycle of *D. plantaginea*, and with the observation of eggs on apple trees during the winter [17], [22]. We found no evidence for the existence of obligate parthenogenesis in *D. plantaginae*, at least on apple trees in the fall and spring. However, it remains possible that anholocyclic lineages exist during these periods of the year on secondary hosts, as reported for many aphid species displaying host alternation [42].

The populations sampled in the fall, before the occurrence of recombination, were produced by lineages that had gone through several parthenogenetic generations since the last sexual event. We therefore expected to find genetic signs of clonal reproduction (repeated multilocus genotypes, LD, systematic HW disequilibrium) in the samples collected in the fall. However, no such signs were observed. This suggests that a single yearly sexual reproduction event is sufficient to generate a high level of genetic variability and to cancel out the genetic signs of clonality, even in the fall, before the occurrence of sexual reproduction. The almost entire absence of individuals with identical multilocus genotypes in samples collected in the fall suggests that the number of individuals from an individual clone of *D. plantaginea* present on apple trees in France in the fall is not large. This may be due to 1) the limited size of the clonal populations sharing the same genotype on secondary hosts compared to the number of different clonal genotypes present on these plants and/or 2) an extensive geographic redistribution of the aphids during their return flight to their primary hosts (but see below), leading the dilution of repeated clonal genotypes.

The high level of genetic variability found in *D. plantaginea* on its primary host is similar to that found in other cyclic parthenogenetic aphids, such as *M. persicae* in France [24] and Australia [45], *S. avenae* [46] or *R. padi* [23] and other cyclic parthenogenetic animals, such as rotifers (e.g. *Brachionus plicatilis* (Müller), [47]), which display high levels of genotypic diversity despite going through numerous parthenogenetic generations each year.

We frequently observed heterozygote deficits associated with HW disequilibrium. Possible explanations based on previous findings for aphids include a Wahlund effect, null alleles, inbreeding and selection [23], [24], [48], [49], [50].

The Wahlund effect is the unintentional pooling of differentiated populations into a single sample, resulting in excess homozygosity [43]. A Wahlund effect may occur in the fall, due to the co-occurrence on the primary host of migrants originating from populations that were genetically differentiated on secondary hosts. Such genetic differentiation may result from genetic drift or selection (e.g. adaptation to various secondary host plants). Panmictic sexual reproduction leads to HW equilibrium in only one generation [43]. Thus, assuming panmictic sexual reproduction, heterozygote deficits in the spring (i.e. after sexual reproduction) cannot be accounted for by a Wahlund effect.

Null alleles were suspected for three loci, and specific statistical treatments were carried out to take this possibility into account. A specific statistical analysis was carried out to detect loci with null alleles, but we cannot rule out the possibility that this problem occurred at a larger number of loci.

Inbreeding and selection are often proposed as explanations for heterozygote deficits in sexual aphid populations [23], [48], [49], [50], but we found no evidence to support this hypothesis in this study.

### Spatial genetic differentiation

Another key finding of this study was the very weak spatial genetic differentiation between *D. plantaginae* populations. We detected no population genetic differentiation at the regional scale or at the intra-orchard or inter-orchard level, for samples located less than 20 km apart. Classically, spatial genetic differentiation results from the balance between migration and genetic drift [51]. In species with mitotic parthenogenesis, selection at one or a few loci affects allele frequency not only at these loci, but throughout the genome, because there is no recombination. Therefore, in a species like *D. plantaginae*, the use of microsatellites to assess spatial population genetic structure also provides information about selection (until sexual reproduction takes place). Our results therefore suggest that the effect of local drift or selection is largely compensated by migration. The fall and spring flights of the aphids mediating host shift are thus sufficient to homogenize genetic variability at a local and regional scale. However, we observed significant levels of population genetic differentiation at the scale of the entire country (France), between different apple-growing areas, with differences observed between Avignon, Agen, Valence and Angers. This genetic differentiation was weak (*F_ST_* generally below 1%) and no isolation by distance was observed, but these results nonetheless suggest that the emigration and return flights of *D. plantaginae* are limited by geographic distance, at regional scale at least, in France. *D. plantaginea* has only one winter host-plant, apple, and this species has a patchy distribution in France. This may account for the spatial limitation of migration. We also found evidence for a local dispersion component in the fall and spring. The sharing of the same multilocus genotype by a pair of individuals on the primary host in the fall, before sexual reproduction, was rare, but nonetheless observed in three instances. In each case, the two individuals sharing the same MLG were found in the same orchard. This strongly suggests that the return flight was local. In other words, this migration may connect secondary and primary hosts located close together, rather than reflecting global geographic homogenization.

The situation in spring was similar to that in the fall and provides information about dispersal between primary hosts after sexual reproduction: three repeated MLGs, each shared by a single pair of individuals, were observed in three different orchards, among 118 colonies. One of the repeated MLGs corresponded to individuals collected from the same tree on two different dates and, thus, probably reflected sampling from the same aphid colony. However, in the two other cases of aphids sharing other repeated MLGs, individuals were collected from non contiguous trees, probably reflecting the dispersion of aphids between different trees in the spring. It is unknown whether such dissemination between distant trees is passive (through wind or cropping practices) or active. Overall, these findings suggest that, at the time of sampling in May, i) aphid dispersal between primary hosts occurred but was not frequent and/or ii) dispersion may have been frequent but only a small proportion of the total number of colonies was sampled. A rough estimate of the sampling effort in spring would be one colony sampled per five actual colonies, so the probability of sampling the same MLG twice or more was low.

Overall, spatial genetic differentiation in *D. plantaginea* was very weak or null over short distances and weak but significant over large distances, suggesting that local migration occurs in<-- --> *D. plantaginea*. This situation is similar to that reported for other aphid species. For instance, in *R. padi*, no genetic differentiation was found between populations located less than 1000 km apart [23]. Weak population differentiation was found between both close (<100 km) and distant (>500 km) populations of the cereal aphid, *S. avenae* [48], [52], [53]. This work provides an additional demonstration that genetic differentiation is not rare in aphids and that aphid migration probably therefore occurs over limited spatial areas [24], [48], [53], [54], [55], [56].

### Temporal genetic differentiation

The third key result of this study is the almost complete temporal genetic homogeneity among samples. Only very low levels of genetic differentiation were observed between samples collected in fall 2006, spring 2007 and fall 2007. There was thus no decrease in genetic variability between the sampling periods. Between the two first sampling periods, one phase of sexual reproduction occurred and a few clonal generations were produced on the primary host. After sexual reproduction on apple trees, *D. plantaginea* is frequently subject to strong demographic bottlenecks, due to pest management practices (e.g. insecticide treatments [5], [6]). In our study system, eight of the 13 orchards were treated conventionally with pesticides. If resistance genes are present in the treated populations, then such pesticide treatments may generate strong selection pressure, increasing the frequency of resistance genes in the clonal aphid population during spring. As recombination is absent during this part of the life cycle, we would expect (i) a change in microsatellite allelic frequencies due to the complete linkage between neutral genetic markers and genes subject to selection and (ii) a decrease in genetic variability due to the increase in frequency of some adapted MLGs. No such change was observed. Moreover, almost no repeated multilocus genotypes potentially resulting from the selection of a few adapted clones were observed in spring. Conventional apple orchards undergo a large number of pesticide treatments (up to 10 treatments are commonly applied in apple orchards when *D. plantaginea* is present, in France [57], and elsewhere see e.g. Blommers *et al.* [18]). Thus, the selection pressure resulting from pesticide treatments is likely to be very intense. Our observation is therefore more consistent with an absence of adaptive gene polymorphism, particularly for insecticide resistance genes, in the populations sampled, the resistance alleles being either fixed or absent. No failure of insecticide treatment was reported in spring 2007, suggesting that the mechanisms of insecticide resistance mechanisms documented by Delorme [58] did not occur.

Using a similar temporal sampling scheme for the peach potato aphid, *Myzus persicae*, Guillemaud *et al*. [24] detected a change in insecticide resistance allele frequency in holocyclic populations in southern France. The *kdr* mutation, which confers resistance to pyrethroid insecticides, increased in frequency between autumn and spring, probably because of insecticide treatments. Conversely, the *rdl* mutation, which confers resistance to cyclodiene insecticides, decreased in frequency over the same period, probably because of the negative pleiotropic effects of the mutation [24].

We also found almost no differentiation between spring 2007 and autumn 2007, a period of time spanning a few clonal generations on the primary host, the emigration flight to secondary hosts followed by a sequence of several clonal generations and the return flight to the apple tree. Again, no decrease in genetic variability was observed between the two sampling points, suggesting that selection and/or drift during the asexual phase of the life cycle has little or no effect on the genetic structure of *D. plantaginea* . This contrasts sharply with what was reported for *M. persicae* by Vorburger [25] and by Guillemaud *et al*. [24], who analyzed changes in population genetic structure during the asexual phase. Vorburger [25] followed the temporal dynamics of *M. persicae* clones on secondary hosts in detail over a period of one year, and Guillemaud *et al*. [24] measured the differentiation between aphids collected during emigration and the return flight. Both studies revealed significant temporal variation of the structure of the population, interpreted in both cases as a result of selection rather than genetic drift. Selection in aphids is now well documented, and it appears that host plant [59], [60], [61], [62] and pesticide treatment [62], [63] are among the most important selective factors to be taken into account when trying to understand the population genetic structure of aphid species acting as crop pests.

No such selective forces appear to shape the population genetic structure of *D. plantaginea* during the asexual phase, which occurs mostly on secondary hosts. The known secondary hosts of *D. plantaginea* are herbaceous plants of the genus *Plantago* [18]. Little is known about possible environmental selection on these plants. No control treatments (such as pesticide applications) are used against *D. plantaginea* when feeding on *Plantago* spp. because these plants are of neither economic nor ornamental value. However, we cannot exclude the possibility that, during the summer, *D. plantaginea* is exposed to pesticides applied to crops or vegetation stands in which their *Plantago* spp. host plants are common (e.g. as weeds). We tried to sample *D. plantaginea* on *Plantago* close to the primary host sampling locations at Valence, without success. This may be because (i) the populations of *D. plantaginea* on the secondary host are small, (ii) secondary host colonization is restricted to particular *Plantago* populations or to plants growing under specific favorable conditions or (iii) *Plantago* is not the only secondary host of *D. plantaginae*. It may be important to identify the entire set of actual secondary host plants of *D. plantaginea* and their distribution, to determine which processes may occur during the asexual phase on the secondary host plant (currently seen as a “black-box”).

### Practical aspects of aphid management

Our results concerning the genetic structure of the rosy apple aphid population have practical implications for the management of this aphid. We found no genetic differences between samples collected from orchards planted with different cultivars (Ariane, Smoothee and Melrose; unfortunately we could not test for an effect of pesticide treatments in Valence in spring 2007 because the sample size was too small for low-input and conventional orchards). There are three possible explanations for this result: (i) None of the three apple cultivars was thought to be resistant to the rosy apple aphid, so there is probably no adaptation to these cultivars in *D. plantaginea*. (ii) Determination of the genetic structure of the population with microsatellites does not reveal genetic structure due to selection, because recombination during sexual reproduction breaks the linkage between adaptive alleles and microsatellite markers. (iii) Migration homogenizes genotypic frequencies, so it is not possible to determine the genetic structure of the population linked to selective forces. The balance between migration and selection was in favor of migration, as discussed below.

We found that migration had a larger effect than drift and selection in shaping the population genetic structure of this species at various geographic scales. The imbalance in favor of migration was found within orchards, between orchards separated by tens of meters at the same site and between sites separated by one to several hundreds of kilometers. This imbalance has two consequences: local adaptation [64] probably cannot occur, and adaptations to control practices may spread rapidly over large geographic areas. Local adaptation may occur when the environment is heterogeneous for selection (e.g. with or without pesticide treatment) and when a there is cost associated with adaptation (e.g. a cost to pesticide resistance). It occurs when a mutated genotype (e.g. a pesticide-resistant genotype) is better adapted to certain local conditions (e.g. pesticide application) but less well adapted to other environmental conditions (e.g. absence of pesticide treatment) than the wild-type genotypes (e.g. pesticide-susceptible genotypes). Management strategies, such as treatment applications limited to small geographic pockets (the stable zone strategy in [65]), based on local adaptations may therefore not be applicable for the rosy apple aphid on apple trees in France. The second consequence of the apparently extensive migration of the rosy apple aphid is that a monogenic or oligogenic genotype adapted to control strategies (e.g. pesticide-resistant genotypes or genotypes circumventing plant resistance) may invade large areas very rapidly after its emergence. This is a potential Achilles heel of control strategies against *D. plantaginea*, because adaptation at any one site may lead to the failure of control everywhere. Resistance to carbamate and organophosphate insecticides has recently been found in a *D. plantaginea* clone collected in Avignon (Southern France) [58]. This resistance is probably oligenic and based on a small number of biochemical mechanisms. Our results suggest that it is likely to increase rapidly in frequency and spread geographically, leading to the failure of pest control over large areas if no other pesticides (such as pyrethroids) are used.

## Supporting Information

Table S1 **Description of** ***Dysaphis plantaginea*** **samples and genetic variation within samples.**
(DOC)
