# Supplementary material for: Weak Spatial and Temporal Population Genetic Structure in the Rosy Apple Aphid, Dysaphis plantaginea, in French Apple Orchards
Source: PLoS One. 2011 Jun 20;6(6):e21263. doi: 10.1371/journal.pone.0021263 (PMC3119056; doi:10.1371/journal.pone.0021263)
Supplement: Table S1 — Description of Dysaphis plantaginea samples and genetic variation within samples. (DOC) [file pone.0021263.s001.doc]

**Table S1**: Description of *Dysaphis plantaginea* samples and genetic variation within samples. Lat and Long are the latitude and longitude of the sample location, respectively (WGS 84). Cult is the apple tree cultivar, and Mode is the pesticide treatment regime (org and conv for organic and conventional, respectively). *N* is the number of individuals analyzed, *Na* is the mean number of alleles per locus, *Ho* and *He* are the observed and expectected heterozygosities, respectively. *HW p*-value is the *p*-value for the Hardy-Weinberg (HW) test, and *LD* is the number of significant linkage disequilibrium (LD) tests. The Bonferroni and Benjamini & Hochberg [39] corrections were applied to LD and HW tests, respectively. *F*_IS_ 5 loci and *HW* 5 loci *p*-value were calculated after the removal of the *DPB10*, *DPL4* and *AF93* loci. The star in the geographic coordinate column indicates approximate values.

| Sampling Period | Location | Orchard | Lat | Long | Cult | Mode | *N* | *Na* | *Ho* | *He* | *F*_IS_ | *HW* | *HW p*-value | *LD* | *F*_IS_ 5 loci | *HW* 5 loci *p*-value |
| --- | --- | --- | --- | --- | --- | --- | --- | --- | --- | --- | --- | --- | --- | --- | --- | --- |
| Autumn 2006 | Angers | D1 | 47.480 | -0.613 | multiple | conv | 16 | 7.00 | 0.51 | 0.66 | 0.23 | 2 | **<0.001** | 0 | 0.05 | 0.84 |
|  |  | P32 | 47,480 | -0,611 | multiple | conv | 24 | 7.98 | 0.53 | 0.62 | 0.16 | 1 | **<0.001** | 0 | -0.02 | 0.63 |
|  | Avignon | 157 | 43,794 | 4,890 | Golden | conv | 55 | 9.50 | 0.56 | 0.62 | 0.10 | 2 | **<0.001** | 2 | 0.04 | 0.37 |
|  |  | 65 | 43,786 | 4,919 | Gala | conv | 53 | 9.63 | 0.56 | 0.64 | 0.14 | 3 | **<0.001** | 3 | 0.04 | 0.46 |
|  |  | INRA | 43,914 | 4,884 | Gala | conv | 30 | 8.13 | 0.57 | 0.60 | 0.06 | 0 | 0.25 | 0 | 0.02 | 0.88 |
|  | Valence | Ariane IPM | 44,980 | 4,925 | Ariane | conv | 7 | 4.63 | 0.61 | 0.63 | 0.04 | 0 | 0.10 | 0 | -0.12 | 0.73 |
|  |  | Smoothee1 (border) | 44.976 | 4.925 | Smoothee | org | 20 | 7.13 | 0.61 | 0.64 | 0.05 | 1 | 0.09 | 0 | -0.01 | 0.64 |
|  |  | (centre) | 44,976 | 4,925 | Smoothee | org | 33 | 7.88 | 0.50 | 0.61 | 0.19 | 2 | **<0.001** | 0 | 0.03 | 0.57 |
| Spring 2007 | Valence | Organic Ariane | 44,979 | 4,925 | Ariane | org | 50 | 10.13 | 0.57 | 0.62 | 0.09 | 2 | **<0.001** | 1 | 0.05 | 0.02 |
|  |  | Low-input Ariane | 44,979 | 4,925 | Ariane | conv | 2 | 2.50 | 0.63 | 0.65 | 0.05 | 0 | 0.93 | 0 | 0.07 | 0.82 |
|  |  | Conventional Ariane | 44,979 | 4.929 | Ariane | conv | 5 | 4.63 | 0.60 | 0.67 | 0.12 | 0 | 0.55 | 0 | 0.14 | 0.38 |
|  |  | Organic Melrose | 44,979 | 4,925 | Melrose | org | 36 | 9.38 | 0.58 | 0.67 | 0.14 | 2 | **0.01** | 5 | 0.04 | 0.40 |
|  |  | Organic Smoothee | 44,979 | 4,925 | Smoothee | org | 25 | 8.75 | 0.57 | 0.65 | 0.13 | 0 | **0.01** | 2 | 0.08 | 0.01 |
| Autumn 2007 | Agen | Agen | 44,391 | 0.586 | Golden | org | 34 | 8.50 | 0.52 | 0.61 | 0.15 | 3 | **<0.001** | 0 | 0.05 | 0.67 |
|  | Angers | D1 | 47.480 | -0.613 | multiple | conv | 23 | 7.75 | 0.57 | 0.63 | 0.09 | 1 | 0.11 | 0 | 0.14 | 0.01 |
|  |  | P32 | 47,480 | -0,611 | multiple | conv | 32 | 9.38 | 0.59 | 0.61 | 0.03 | 1 | 0.10 | 0 | 0.04 | 0.07 |
|  | Avignon | 157 | 43,794 | 4,890 | Golden | conv | 35 | 8.13 | 0.42 | 0.59 | 0.23 | 2 | **<0.001** | 0 | 0.14 | **<0.001** |
|  |  | 65 | 43,786 | 4,919 | Gala | conv | 11 | 4.13 | 0.36 | 0.54 | 0.35 | 1 | **0.00** | 0 | 0.26 | 0.03 |
|  | Valence | Ariane IPM | 44,980 | 4,925 | Ariane | conv | 25 | 7.38 | 0.54 | 0.62 | 0.14 | 1 | **0.01** | 0 | 0.02 | 0.34 |
|  |  | Smoothee1 | 44.976 | 4.925 | Smoothee | org | 16 | 7.13 | 0.55 | 0.65 | 0.16 | 2 | **<0.001** | 0 | 0.12 | 0.04 |
